# Pertuzumab plus trastuzumab and chemotherapy for Japanese patients with HER2-positive metastatic gastric or gastroesophageal junction cancer: a subgroup analysis of the JACOB trial

**DOI:** 10.1007/s10147-019-01558-z

**Published:** 2019-10-16

**Authors:** Kohei Shitara, Hiroki Hara, Takaki Yoshikawa, Kazumasa Fujitani, Tomohiro Nishina, Ayumu Hosokawa, Takashi Asakawa, Satoe Kawakami, Kei Muro

**Affiliations:** 1 Gastroenterology and Gastrointestinal Oncology, National Cancer Center Hospital East, 6-5-1 Kashiwanoha, Kashiwa, Chiba 277-8577 Japan; 2 Department of Gastroenterology, Saitama Cancer Center, 780 Komuro, Ina, Saitama 362-0806 Japan; 3 Department of Gastrointestinal Surgery, Kanagawa Cancer Center, 2-3-2 Nakano, Asahi-ku, Yokohama, 241-8515 Japan; 4 Present Address: Department of Gastric Surgery, National Cancer Center Hospital, 5-1-1 Tsukiji, Chuo-ku, Tokyo, 104-0045 Japan; 5 Department of Surgery, Osaka General Medical Center, 3-1-56 Bandaihigashi Sumiyoshi-ku, Osaka, 558-8558 Japan; 6 Department of Gastrointestinal Medical Oncology, National Hospital Organization Shikoku Cancer Center, Kou-160, Minamiumemoto-machi, Matsuyama, Ehime Japan; 7 Department of Gastroenterology and Hematology, Faculty of Medicine, University of Toyama, 2630 Sugitani, Toyama, Toyama 930-0194 Japan; 8 Present Address: Department of Clinical Oncology, University of Miyazaki Hospital, 5200 Kiyotakecho Kihara, Miyazaki, Miyazaki 889-1692 Japan; 9 Clinical Information and Intelligence Department, Chugai Pharmaceutical Co., Ltd, 2-1-1, Nihonbashi-Muromachi, Chuo-ku, Tokyo, Japan; 10 Clinical Science & Strategy Department, Chugai Pharmaceutical Co., Ltd, 2-1-1, Nihonbashi-Muromachi Chuo-ku, Tokyo, Japan; 11 Department of Clinical Oncology, Aichi Cancer Center Hospital, 1-1 Kanokoden, Chikusa-ku, Nagoya, Japan

**Keywords:** Pertuzumab, Trastuzumab, Metastatic gastric cancer, Metastatic gastroesophageal junction cancer, Japanese subgroup analysis, Phase III

## Abstract

**Background:**

The phase III JACOB trial (NCT01774786) compared the efficacy and safety of pertuzumab and trastuzumab plus chemotherapy with placebo and trastuzumab plus chemotherapy in patients with previously untreated human epidermal growth factor receptor 2 (HER2)-positive metastatic gastric or gastroesophageal junction cancer. We conducted a subgroup analysis in Japanese patients.

**Methods:**

Patients were randomized 1:1 to pertuzumab 840 mg or placebo, plus trastuzumab (loading dose, 8 mg/kg; maintenance dose, 6 mg/kg) and chemotherapy (cisplatin 80 mg/m^2^, and capecitabine 1000 mg/m^2^ twice daily for 28 doses or 5-fluorouracil 800 mg/m^2^ every 24 h for 120 h), every 3 weeks. Continuation of chemotherapy after 6 cycles was at the discretion of the patient and the treating physician.

**Results:**

A total of 40 Japanese patients were included in each arm. Median overall survival was 22.0 months (95% confidence interval [CI] 13.8–not evaluable) and 15.6 months (95% CI 9.7–19.2) in the pertuzumab and placebo arms, respectively (hazard ratio [HR] 0.64 [95% CI 0.37–1.10]). Median progression-free survival was 12.4 months (95% CI 6.1–14.1) in the pertuzumab arm and 6.3 months (95% CI 4.3–8.1) in the placebo arm (HR 0.50 [95% CI 0.30–0.82]). Grade ≥ 3 adverse events and serious adverse events were more frequent in the pertuzumab arm than the placebo arm.

**Conclusions:**

Results from this subgroup analysis of the JACOB trial suggest similar efficacy of pertuzumab in Japanese patients and patients in the overall population, encouraging continued investigation of new agents for gastric cancer in Japanese patients.

**Electronic supplementary material:**

The online version of this article (10.1007/s10147-019-01558-z) contains supplementary material, which is available to authorized users.

## Introduction

The Trastuzumab for Gastric Cancer (ToGA) trial (ClinicalTrials.gov identifier, NCT01041404) demonstrated improvements in overall survival (OS) when trastuzumab was added to chemotherapy, compared with chemotherapy alone, in previously untreated patients with human epidermal growth factor receptor 2 (HER2)-positive advanced or metastatic gastric or gastroesophageal junction cancer [1]. Based on these results, trastuzumab-containing regimens are now standard of care for first-line treatment of this disease [2–4].

Like trastuzumab, pertuzumab is a humanized monoclonal antibody that targets the HER2 receptor, but that binds to a different epitope. Dual HER2 blockade with pertuzumab and trastuzumab, plus chemotherapy, has been shown to improve survival outcomes in patients with HER2-positive early and metastatic breast cancer [5–7].

The phase III JACOB trial (ClinicalTrials.gov identifier, NCT01774786) was designed to compare the efficacy and safety of pertuzumab and trastuzumab plus chemotherapy with placebo and trastuzumab plus chemotherapy in patients with previously untreated HER2-positive metastatic gastric or gastroesophageal junction cancer [8]. The earlier phase IIa JOSHUA trial (ClinicalTrials.gov identifier, NCT01461057) had demonstrated that a pertuzumab dose of 840 mg every 3 weeks led to higher serum trough concentrations versus the currently approved dose for use in HER2-positive metastatic breast cancer (840 mg loading dose/420 mg maintenance dose), thus allowing patients with HER2-positive gastric cancer to achieve pertuzumab serum trough concentrations similar to those observed in patients with HER2-positive metastatic breast cancer, with a safety profile similar to the 840 mg loading dose/420 mg maintenance dose regimen [9, 10]. Therefore, the dose of 840 mg of pertuzumab every 3 weeks was selected for investigation in the JACOB trial.

Results from the JACOB trial showed no statistically significant improvements in OS between the pertuzumab and placebo arms (median 17.5 months [95% confidence interval (CI) 16.2–19.3] and 14.2 months [95% CI 12.9–15.5], respectively; hazard ratio [HR] 0.84 [95% CI 0.71–1.00]; *p* = 0.057) [8], although the magnitude of the treatment effect on OS appeared to be clinically relevant. A trend toward clinically relevant improvements in progression-free survival (PFS) and objective response rate (ORR) in the pertuzumab arm versus the placebo arm was also observed, and safety was comparable between the treatment arms, with no increased cardiac toxicity observed from the increased maintenance dose compared with the approved breast cancer regimen [8].

In Japan, gastric cancer is the most common malignancy and third leading cause of cancer death, with 128,881 new cases reported in 2015 and 45,226 related deaths in 2017 [11, 12]. Some studies have suggested that outcomes for Japanese patients with advanced or metastatic gastric cancer may differ from those in the global population. For example, the ToGA trial demonstrated longer OS in patients from Japan compared with the overall population [13]. In addition, the phase III AVAGAST study (ClinicalTrials.gov identifier, NCT00548548) of bevacizumab plus chemotherapy in advanced gastric cancer reported longer OS in the control arm (placebo plus chemotherapy) and lowered improvement of OS in the bevacizumab arm in patients from Asia compared with those from Eastern Europe/South America and the USA/Western Europe [14, 15]. These differences in survival outcomes may be due to differences in patient baseline characteristics, healthcare, and post-progression treatment across the different regions [14], and highlight the importance of subgroup analyses according to regions. Here, we report results from a subgroup analysis of the JACOB trial to evaluate the efficacy and safety of pertuzumab in combination with trastuzumab and chemotherapy in Japanese patients.

## Patients and methods

### Study design and patients

JACOB was a double-blind, placebo-controlled, randomized phase III trial. Details of the study design have been published previously [8]. Briefly, eligible patients were randomized 1:1 using a stratified permuted block randomization scheme with an interactive voice or web response system (IxRS) to receive pertuzumab 840 mg intravenously or placebo, in combination with intravenous trastuzumab (loading dose, 8 mg/kg; maintenance dose, 6 mg/kg) and chemotherapy (cisplatin 80 mg/m^2^ intravenously plus capecitabine 1000 mg/m^2^ taken orally twice a day for 28 doses or 5-fluorouracil 800 mg/m^2^ every 24 h intravenously by continuous infusion for 120 h), every 3 weeks. Chemotherapy was continued for 6 cycles, except in the event of progressive disease or unacceptable toxicity, and was continued after 6 cycles at the discretion of the patient and the treating physician. The study was performed in accordance with the Declaration of Helsinki and the International Conference on Harmonisation E6 Good Clinical Practice (ICH-GCP-E6) guidelines, or the laws and regulations of the country in which the research was performed, whichever provided the greater protection for the patients. Approval for the study protocol, any protocol amendments, and all material provided to the patients was obtained from the relevant institutional review board or ethics committee at each site, and all patients provided written informed consent.

## Objectives

The primary objective of the JACOB trial was to compare OS in patients treated with pertuzumab plus trastuzumab and chemotherapy with those treated with placebo plus trastuzumab and chemotherapy. Key secondary objectives included comparison of PFS, ORR, duration of response, patient-reported outcomes (PROs), and safety between the two arms.

### Statistical analysis

OS and PFS were assessed in the intention-to-treat (ITT) population (all patients randomly assigned to a treatment group, regardless of whether they received a study drug). ORR was assessed in patients in the ITT population who had measurable disease at baseline, and patient-reported outcomes in patients in the ITT population who had assessments at baseline and at least one assessment after baseline. Safety was assessed in all randomly assigned patients who had received at least one dose of study treatment.

The Kaplan–Meier method was used to estimate the distributions of OS, PFS, and duration of response, including estimates of the medians, with 95% CIs calculated using the Brookmeyer and Crowley method. Cox proportional hazards regression models were used to estimate the HR for OS and PFS with 95% CIs between treatment arms. For ORR and clinical benefit rate, the proportion of patients achieving an overall response per the Response Evaluation Criteria In Solid Tumors (RECIST) version 1.1 was summarized and 95% CIs were calculated using the Clopper–Pearson method. Adverse events (AEs) were graded per the National Cancer Institute – Common Terminology Criteria for Adverse Events (NCI-CTCAE), version 4.03. PRO assessments included health-related quality of life and time to deterioration (TTD) in gastric cancer-related symptoms, which were assessed using the European Organisation for Research and Treatment of Cancer (EORTC) Quality of Life Questionnaire of cancer patients (QLQ-C30 v3.0), gastric cancer module (QLQ-STO22), and EuroQol five-dimensional questionnaire (EQ-5D-3L). TTD assessments included the time from baseline to ≥ 10-point increase in abdominal pain, eating restriction, appetite loss, and fatigue (TTD1), and the TTD from initiation of therapy with pertuzumab/placebo plus trastuzumab alone following cessation of chemotherapy (TTD2). The distribution of TTD, including an estimate of the median, was assessed using the Kaplan–Meier method.

## Results

### Patients and treatment

**Fig. 1:**
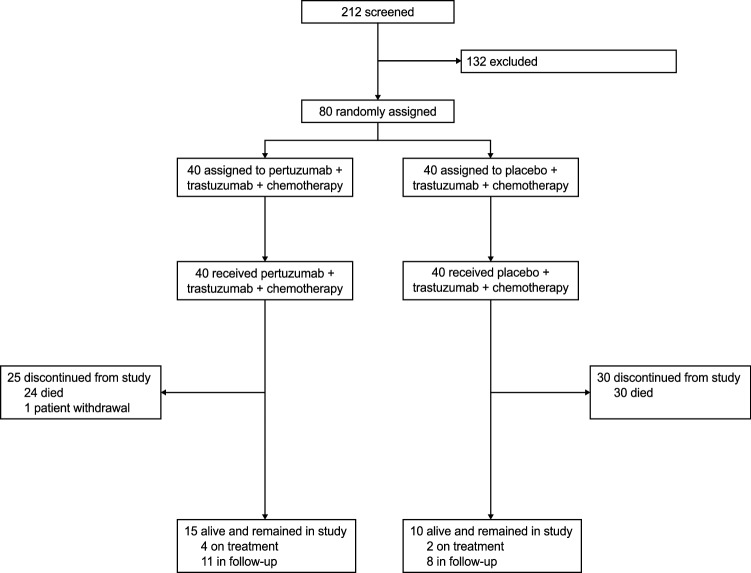
Trial profile (CONSORT diagram)

In total, 80 patients were randomized across 15 centers in Japan between 10 June 2013 and 12 Jan 2016; 40 to each arm. Patient dispositions are shown in Fig. 1. All patients were included in the ITT population and were also evaluated for safety. Baseline patient demographics and disease characteristics are shown in Table 1. Due to the stratified randomization of patients, no major imbalance was observed between arms. Numerically more patients in the pertuzumab arm were male, had non-measurable evaluable disease only, had more than two metastatic sites, had gastroesophageal junction cancer, and had an Eastern Cooperative Oncology Group performance status of 0, compared with the placebo arm. In the majority of patients in both arms, the histologic subtype was intestinal and the primary site was the stomach. Nearly two-thirds of patients in each arm had a HER2 immunohistochemistry status of 3 +.

**Table 1:** Baseline demographics and disease characteristics (ITT population, Japanese subgroup)

	Pertuzumab (*n* = 40)	Placebo (*n* = 40)
Sex, *n* (%)		
Male	33 (82.5)	28 (70.0)
Female	7 (17.5)	12 (30.0)
Median age, years (range)	68.5 (36–78)	70.0 (53–82)
Measurability, *n* (%)		
Measurable disease	34 (85.0)	37 (92.5)
Non-measurable evaluable disease only	6 (15.0)	3 (7.5)
Number of metastatic sites, *n* (%)		
1–2	31 (77.5)	35 (87.5)
> 2	9 (22.5)	5 (12.5)
Histologic subtypes,^a^*n* (%)		
Diffuse	1 (2.5)	1 (2.5)
Intestinal	38 (95.0)	36 (90.0)
Mixed	1 (2.5)	3 (7.5)
Primary site, *n* (%)		
Gastroesophageal junction	7 (17.5)	4 (10.0)
Stomach	33 (82.5)	36 (90.0)
ECOG performance status, *n* (%)		
0	31 (77.5)	25 (62.5)
1	9 (22.5)	15 (37.5)
HER2 status, *n* (%)		
IHC 2 + / ISH +	14 (35.0)	15 (37.5)
IHC 3 +	26 (65.0)	25 (62.5)
Previous gastrectomy, *n* (%)		
Yes	6 (15.0)	5 (12.5)
No	34 (85.0)	35 (87.5)

*ECOG* Eastern Cooperative Oncology Group, *HER2* human epidermal growth factor receptor 2, *IHC *immunohistochemistry, *ISH* in situ hybridization, *ITT* intention-to-treat

^a^Histologic subtypes are based on Lauren classification criteria

At the clinical cutoff date (December 9, 2016), the median duration of follow-up was 33.2 months (95% CI 31.3–35.5) in the pertuzumab arm and 34.0 months (95% CI 31.5–36.3) in the placebo arm. Study treatment exposure is shown in Table 2. The median number of pertuzumab/placebo and trastuzumab treatment cycles per patient was higher in the pertuzumab arm compared with the placebo arm (14 [range: 1–45] vs 8 [range: 1–51] cycles). The median relative dose intensity for pertuzumab/placebo and trastuzumab was comparable in the two treatment arms. For capecitabine, the median number of treatment cycles was comparable between treatment arms and the median relative dose intensity was slightly lower in the pertuzumab arm compared with the placebo arm. The median number of cisplatin treatment cycles and the median relative dose intensity for cisplatin were both comparable between treatment arms. No patients in the Japanese subgroup received 5-fluorouracil. The number of patients who received at least one post-treatment cancer therapy during the study was 28 (70.0%) and 31 (77.5%) in the pertuzumab and placebo arms, respectively (Table S1).

**Table 2:** Study treatment exposure (safety population)

	Relative dose intensity^a^ (%)				Number of cycles per patient			
	Pertuzumab (*n* = 40)		Placebo (*n* = 40)		Pertuzumab (*n* = 40)		Placebo (*n* = 40)	
	Median (range)	Mean (SD)	Median (range)	Mean (SD)	Median (range)	Mean (SD)	Median (range)	Mean (SD)
Pertuzumab/placebo	87.9 (75–100)	88.0 (7.3)	88.7 (62–100)	88.6 (9.4)	14 (1–45)	16.13 (12.24)	8 (1–51)	9.65 (9.99)
Trastuzumab	85.1 (63–110)	85.4 (10.4)	88.1 (68–104)	87.8 (9.3)	14 (1–45)	16.13 (12.24)	8 (1–51)	9.65 (9.99)
Capecitabine	54.4 (22–99)	57.6 (17.9)	69.4 (38–100)	68.2 (16.5)	7 (1–42)	11.10 (9.71)	6 (1–51)	8.38 (9.84)
5-fluorouracil	–	–	–	–	–	–	–	–
Cisplatin	73.0 (41–101)	72.5 (18.9)	75.5 (50–101)	76.8 (17.0)	5 (1–10)	4.68 (2.21)	6 (1–6)	4.45 (2.02)

*SD* standard deviation

^a^Relative dose intensity is defined as the percentage of the actual delivered dose intensity divided by the standard dose intensity

### Efficacy

**Fig. 2:**
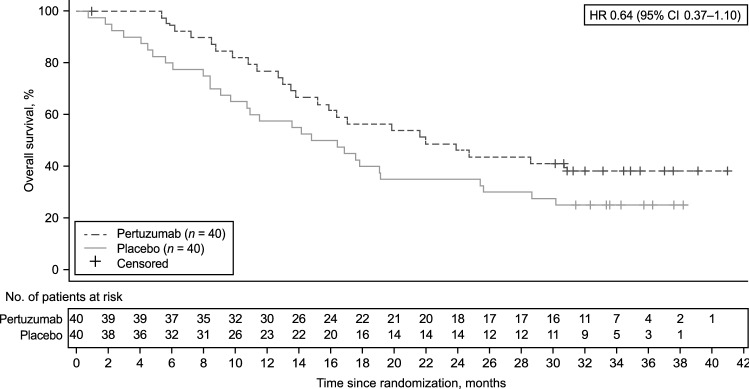
Overall survival in the ITT population. *CI* confidence interval, *HR* hazard ratio

**Fig. 3:**
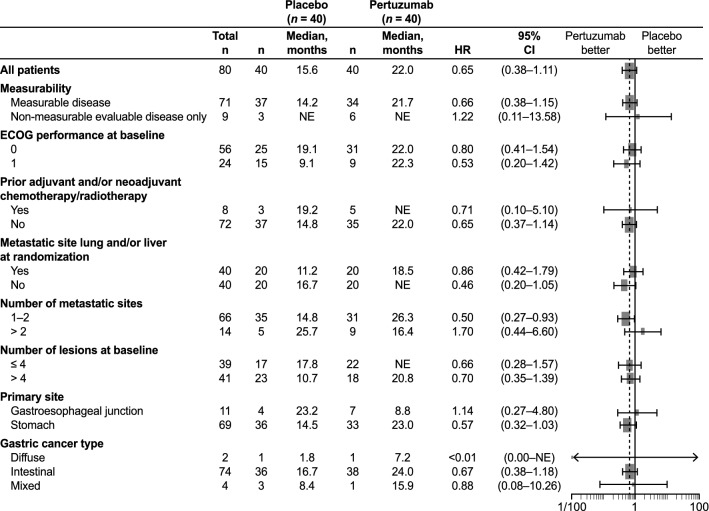
Subgroup analysis of overall survival. *CI* confidence interval, *ECOG* Eastern Cooperative Oncology Group *HR*, hazard ratio, *NE* not evaluable

**Fig. 4:**
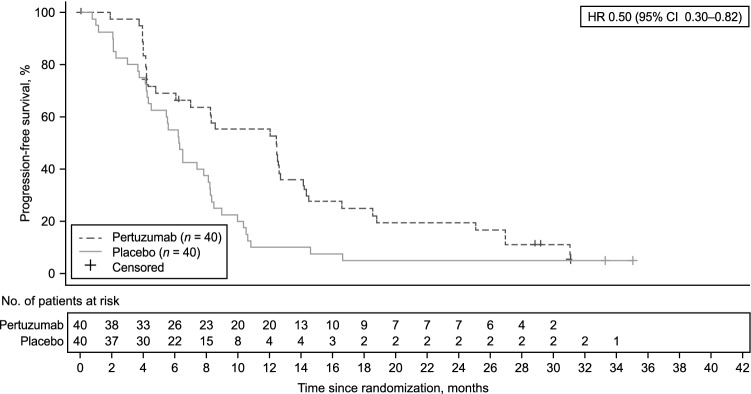
Progression-free survival in the ITT population. *CI* confidence interval, *HR* hazard ratio

A total of 24 (60.0%) patients in the pertuzumab arm and 30 (75.0%) patients in the placebo arm had died on-study at the time of data cutoff. Median OS was 22.0 months (95% CI 13.8–not evaluable) in the pertuzumab arm and 15.6 months (95% CI 9.7–19.2) in the placebo arm (HR 0.64 [95% CI 0.37–1.10]) (Fig. 2). Similar results were observed for OS in the majority of the subgroup analyses (Fig. 3). Median PFS was 12.4 months (95% CI 6.1–14.1) in the pertuzumab arm and 6.3 months (95% CI 4.3–8.1) in the placebo arm (Fig. 4). A trend toward an improvement in PFS was observed in the pertuzumab arm compared with the placebo arm (HR 0.50 [95% CI 0.30–0.82]). There was also a trend toward higher ORR in the pertuzumab arm (61.8% [95% CI 43.56–77.83]) than in the placebo arm (40.5% [95% CI 24.75–57.90]). No patients achieved a complete response.

### Safety

All patients included in the study experienced AEs. Table 3 shows AEs occurring in ≥ 20% of patients at any grade in either arm, by grade. The most common AEs of any grade were decreased appetite (*n* = 35 [87.5%]), diarrhea (*n* = 34 [85.0%]), nausea (*n* = 29 [72.5%]), palmar–plantar erythrodysesthesia (*n* = 29 [72.5%]), neutropenia (*n* = 27 [67.5%]), and fatigue (*n* = 25 [62.5%]) in the pertuzumab arm, and decreased appetite (*n* = 33 [82.5%]), palmar–plantar erythrodysesthesia (*n* = 25 [62.5%]), and nausea (*n* = 24 [60.0%]) in the placebo arm. The majority of the most common any-grade AEs occurred more frequently in the pertuzumab arm compared with the placebo arm. Grade ≥ 3 AEs were reported in 38 (95.0%) patients in the pertuzumab arm and 30 (75.0%) patients in the placebo arm. The most common grade ≥ 3 AEs were decreased appetite (*n* = 19 [47.5%]), neutropenia (*n* = 20 [50.0%]), and diarrhea (*n* = 9 [22.5%]) in the pertuzumab arm, and decreased appetite (*n* = 11 [27.5%]), neutropenia (*n* = 12 [30.0%]), and hyponatremia (*n* = 8 [20.0%]) in the placebo arm. Serious AEs occurred in 27 (67.5%) patients and 13 (32.5%) patients in the pertuzumab and placebo arms, respectively. Two (5.0%) patients in the pertuzumab arm and four (10.0%) patients in the placebo arm had AEs that led to treatment discontinuation; these AEs were decreased ejection fraction and muscle weakness (*n* = 1 each) in the pertuzumab arm and decreased ejection fraction, multiple organ dysfunction syndrome, gastrointestinal anastomotic leak, and renal dysfunction (*n* = 1 each) in the placebo arm. The total number of deaths was 24 (60.0%) in the pertuzumab arm and 30 (75.0%) in the placebo arm, of which one in each arm was AE-related. Fatal AEs were myocardial infarction (*n* = 1) in the pertuzumab arm and multiple organ dysfunction syndrome (*n* = 1) in the placebo arm. A summary of cardiac AEs is provided in Table 4.

**Table 3:** AEs occurring in ≥ 20% of patients at any grade in either arm, by grade

AEs by preferred term,^a^*n* (%)	Pertuzumab (*n* = 40)					Placebo (*n* = 40)				
	Any grade	Grade 1–2	Grade 3	Grade 4	Grade 5	Any grade	Grade 1–2	Grade 3	Grade 4	Grade 5
Nausea	29 (72.5)	23 (57.5)	6 (15.0)	0	0	24 (60.0)	20 (50.0)	4 (10.0)	0	0
Diarrhea	34 (85.0)	25 (62.5)	9 (22.5)	0	0	15 (37.5)	13 (32.5)	2 (5.0)	0	0
Stomatitis	18 (45.0)	16 (40.0)	2 (5.0)	0	0	20 (50.0)	19 (47.5)	1 (2.5)	0	0
Constipation	12 (30.0)	12 (30.0)	0	0	0	18 (45.0)	18 (45.0)	0	0	0
Vomiting	14 (35.0)	13 (32.5)	1 (2.5)	0	0	10 (25.0)	10 (25.0)	0	0	0
Fatigue	25 (62.5)	23 (57.5)	2 (5.0)	0	0	14 (35.0)	13 (32.5)	1 (2.5)	0	0
Edema	12 (30.0)	12 (30.0)	0	0	0	17 (42.5)	17 (42.5)	0	0	0
Fever	18 (45.0)	17 (42.5)	1 (2.5)	0	0	9 (22.5)	9 (22.5)	0	0	0
Malaise	9 (22.5)	9 (22.5)	0	0	0	12 (30.0)	12 (30.0)	0	0	0
Chills	11 (27.5)	11 (27.5)	0	0	0	2 (5.0)	2 (5.0)	0	0	0
Peripheral edema	2 (5.0)	2 (5.0)	0	0	0	8 (20.0)	8 (20.0)	0	0	0
Palmar–plantar erythrodysesthesia	29 (72.5)	27 (67.5)	2 (5.0)	0	0	25 (62.5)	25 (62.5)	0	0	0
Dry skin	9 (22.5)	9 (22.5)	0	0	0	8 (20.0)	8 (20.0)	0	0	0
Decreased appetite	35 (87.5)	16 (40.0)	19 (47.5)	0	0	33 (82.5)	22 (55.0)	11 (27.5)	0	0
Hyponatremia	5 (12.5)	1 (2.5)	4 (10.0)	0	0	8 (20.0)	0	8 (20.0)	0	0
Neutropenia	27 (67.5)	7 (17.5)	15 (37.5)	5 (12.5)	0	21 (52.5)	9 (22.5)	8 (20.0)	4 (10.0)	0
Nasopharyngitis	12 (30.0)	12 (30.0)	0	0	0	7 (17.5)	7 (17.5)	0	0	0
Taste disorders	18 (45.0)	18 (45.0)	0	0	0	13 (32.5)	13 (32.5)	0	0	0
Peripheral sensory neuropathy	8 (20.0)	8 (20.0)	0	0	0	7 (17.5)	7 (17.5)	0	0	0
Hiccups	19 (47.5)	19 (47.5)	0	0	0	16 (40.0)	16 (40.0)	0	0	0
Creatinine urine decreased	20 (50.0)	17 (42.5)	3 (7.5)	0	0	11 (27.5)	11 (27.5)	0	0	0
Injection-related reaction	9 (22.5)	9 (22.5)	0	0	0	4 (10.0)	4 (10.0)	0	0	0
Insomnia	10 (25.0)	10 (25.0)	0	0	0	11 (27.5)	11 (27.5)	0	0	0

*AE* adverse event

^a^For frequency counts by preferred term, multiple occurrences of the same AE in an individual are counted only once

**Table 4:** Cardiac safety

	Pertuzumab (*n* = 40)	Placebo (*n* = 40)
Patients with symptomatic LVSD, *n* (%)		
NYHA class III or IV	0	0
Median baseline LVEF (range)	66.0 (55–79)	66.5 (60–78)
Worst post-baseline LVEF and change from baseline, *n* (%)	*n* = 39	*n* = 36
Patients with decrease from baseline of ≥ 10% and to < 50%	2 (5.1)	4 (11.1)

*LVEF* left ventricular ejection fraction, *LVSD* left ventricular systolic dysfunction, *NYHA* New York Heart Association

### PROs

**Fig. 5:**
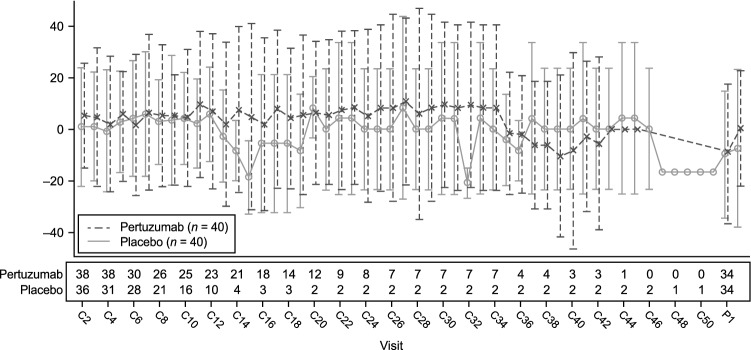
Patient-reported outcomes, mean change from baseline in Global Health Status scale (EORTC QLQ-C30). Error bars represent standard deviations. Higher scores represent higher levels of functioning. *C* cycle, *P* post-treatment assessment

Overall health-related quality of life was similar between arms for the greater part of the study, with no clinically meaningful differences detected (Fig. 5). The TTD1 and TTD2 for abdominal pain, appetite loss, eating restrictions, and fatigue are summarized in Table 5. A consistent trend of improved TTD was observed for each symptom in the pertuzumab arm compared with the placebo arm.

**Table 5:** Patient-reported outcomes: times to deterioration

TTD1	Pertuzumab	Placebo	Stratified HR (95% CI)
	*n* = 40	*n* = 40	
Abdominal pain^a^			0.38 (0.14–1.05)
Patient with events, *n* (%)	6 (15.0)	11 (27.5)	
Median TTD1, months (95% CI)	NE (NE–NE)	NE (7.294–NE)	
Appetite loss symptom scale^b^			0.89 (0.45–1.78)
Patient with events, *n* (%)	18 (45.0)	16 (40.0)	
Median TTD1, months (95% CI)	NE (2.891–NE)	10.842 (1.971–NE)	
Eating restrictions^a^			0.85 (0.43–1.69)
Patient with events, *n* (%)	17 (42.5)	16 (40.0)	
Median TTD1, months (95% CI)	NE (4.107–NE)	9.265 (2.924–NE)	
Fatigue symptom scale^b^			0.58 (0.32–1.02)
Patient with events, *n* (%)	24 (60.0)	27 (67.5)	
Median TTD1, months (95% CI)	4.961 (1.840–NE)	1.873 (1.380–3.220)	

TTD2	*n* = 19	*n* = 11	
Abdominal pain^a^			0.79 (0.13–4.86)
Patient with events, *n* (%)	3 (15.8)	2 (18.2)	
Median TTD2, months (95% CI)	NE (7.984–NE)	NE (1.873–NE)	
Appetite loss symptom scale^b^			0.31 (0.05–1.99)
Patient with events, *n* (%)	3 (15.8)	3 (27.3)	
Median TTD2, months (95% CI)	NE (7.622–NE)	NE (0.789–NE)	
Eating restrictions^a^			0.48 (0.06–3.71)
Patient with events, *n* (%)	3 (15.8)	2 (18.2)	
Median TTD2, months (95% CI)	NE (NE–NE)	NE (2.563–NE)	
Fatigue symptom scale^b^			0.76 (0.19–3.05)
Patient with events, *n* (%)	9 (47.4)	4 (36.4)	
Median TTD2, months (95% CI)	6.932 (0.821–NE)	5.585 (0.789–NE)	

Deterioration is defined as score increase of ≥ 10 points for at least two consecutive cycles or an initial score increase of ≥ 10 points followed by death within 3 weeks

*CI* confidence interval, *EORTC QLQ-C30* European Organisation for Research and Treatment of Cancer Quality of Life Questionnaire of cancer patients, *EORTC QLQ-STO22* European Organisation for Research and Treatment of Cancer Quality of Life Questionnaire gastric cancer module, *HR* hazard ratio, *NE* not evaluable, *TTD1* time from baseline to ≥ 10-point increase in abdominal pain, eating restriction, appetite loss, and fatigue, *TTD2* time to deterioration from initiation of therapy with pertuzumab/placebo plus trastuzumab alone following cessation of chemotherapy

^a^Abdominal pain and eating restrictions assessed using the EORTC QLQ-STO22 questionnaire

^b^Appetite loss and fatigue symptoms assessed using the EORTC QLQ-C30 questionnaire

## Discussion

In this subgroup analysis of Japanese patients in the phase III JACOB trial, efficacy results were in line with those observed for the overall population [8]. However, median OS in Japanese patients (22.0 months [95% CI 13.8–not evaluable] in the pertuzumab arm and 15.6 months [95% CI 9.7–19.2] in the placebo arm) was relatively longer compared with the overall population (17.5 months [95% CI 16.2–19.3] in the pertuzumab arm and 14.2 months [95% CI 12.9–15.5] in the placebo arm) [8]. This is consistent with results from the ToGA trial and from the placebo arm of the AVAGAST trial, which showed improved OS in Japanese patients compared with the overall population, and in Asian patients compared with patients from other regions, respectively [13, 14].

The relatively longer OS observed in Japanese patients compared with the overall population in JACOB could be explained by the following. First, there was a higher proportion of patients with an Eastern Cooperative Oncology Group performance status of 0 (pertuzumab arm: 77.5% in the Japanese subgroup vs 42% in the overall population; placebo arm: 62.5% in the Japanese subgroup vs 41% in the overall population). Second, there was a higher proportion of patients who received post-treatment cancer therapy (pertuzumab arm: 70.0% in the Japanese subgroup vs 42.5% in the overall population; placebo arm: 77.5% in the Japanese subgroup vs 42.1% in the overall population) in the Japanese subgroup compared with the overall population in JACOB. Despite this, our results suggest similar treatment effects of pertuzumab in both the Japanese subgroup and the overall population, encouraging the continuing development of new agents for gastric cancer in Japanese patients.

With regards to safety, the most common any-grade AE and grade ≥ 3 AE in Japanese patients was decreased appetite; in the overall JACOB population, the most common any-grade AE and grade ≥ 3 AE were diarrhea and neutropenia, respectively. The Japanese subgroup experienced a higher frequency of grade ≥ 3 AEs (95.0% vs 80%) and serious AEs (67.5% vs 45%) in the pertuzumab arm compared with the overall population [8]. This was true for each of the most common grade ≥ 3 AEs in the pertuzumab arm in the Japanese subgroup: decreased appetite (47.5% vs 10% in the overall population), neutropenia (50.0% vs 31% in the overall population), and diarrhea (22.5% vs 14% in the overall population) [8]. Similarly increased frequencies of any-grade decreased appetite, neutropenia, and diarrhea in the Asian population compared with the overall population have also been observed in patients with metastatic breast cancer who were treated with pertuzumab plus trastuzumab and chemotherapy in the phase III, randomized, placebo-controlled CLEOPATRA trial [16]. No symptomatic left ventricular systolic dysfunction was reported in Japanese patients, and more patients in the placebo arm experienced left ventricular ejection fraction decreases from baseline of ≥ 10% and to < 50% (although the number of patients was small, and therefore, firm conclusions cannot be drawn).

Limitations of the JACOB trial have been discussed previously [8]. Additional limitations associated with this subgroup analysis include the exploratory nature of the analyses and the small number of patients included in the Japanese subgroup.

In conclusion, the results from this subgroup analysis of the JACOB trial suggest similar efficacy of pertuzumab in Japanese patients and patients in the overall population, encouraging continued investigation of new agents for the treatment of gastric cancer in Japanese patients.

## Electronic supplementary material

Below is the link to the electronic supplementary material.

Supplementary file1 (DOCX 49 kb)

## Data Availability

Qualified researchers may request access to individual patient level data through the clinical study data request platform (www.clinicalstudydatarequest.com). For further details on Chugai’s Data Sharing Policy and how to request access to related clinical study documents, see here (www.chugai-pharm.co.jp/english/profile/rd/ctds_request.html).

## References

[1] Bang YJ, Van Cutsem E, Feyereislova A (2010). Trastuzumab in combination with chemotherapy versus chemotherapy alone for treatment of HER2-positive advanced gastric or gastro-oesophageal junction cancer (ToGA): a phase 3, open-label, randomised controlled trial. Lancet.

[2] Smyth EC, Verheij M, Allum W (2016). Gastric cancer: ESMO clinical practice guidelines for diagnosis, treatment and follow-up. Ann Oncol.

[3] National Comprehensive Cancer Network (NCCN) (2017) NCCN Clinical Practice Guidelines in Oncology (NCCN Guidelines^®^): Gastric Cancer. Version 2.2019. www.nccn.org. Accessed 1 July 2019

[4] Japanese Gastric Cancer Association (2017). Japanese gastric cancer treatment guidelines 2014 (ver. 4). Gastric Cancer.

[5] von Minckwitz G, Procter M, de Azambuja E (2017). Adjuvant pertuzumab and trastuzumab in early HER2-positive breast cancer. N Engl J Med.

[6] Baselga J, Cortés J, Kim SB (2012). Pertuzumab plus trastuzumab plus docetaxel for metastatic breast cancer. N Engl J Med.

[7] Swain SM, Baselga J, Kim SB (2015). Pertuzumab, trastuzumab, and docetaxel in HER2-positive metastatic breast cancer. N Engl J Med.

[8] Tabernero J, Hoff PM, Shen L (2018). Pertuzumab plus trastuzumab and chemotherapy for HER2-positive metastatic gastric or gastro-oesophageal junction cancer (JACOB): final analysis of a double-blind, randomised, placebo-controlled phase 3 study. Lancet Oncol.

[9] Kang YK, Rha SY, Tassone P (2014). A phase IIa dose-finding and safety study of first-line pertuzumab in combination with trastuzumab, capecitabine and cisplatin in patients with HER2-positive advanced gastric cancer. Br J Cancer.

[10] Cortés J, Swain SM, Kudaba I (2013). Absence of pharmacokinetic drug-drug interaction of pertuzumab with trastuzumab and docetaxel. Anticancer Drugs.

[11] Cancer Registry and Statistics, Cancer Information Service, National Cancer Center, Japan (2019) Cancer prevalence data based on local cancer registration national estimates 1975–2015. Published May 2019. https://ganjoho.jp/reg_stat/statistics/dl/index.html. Accessed 2 Aug 2019

[12] Cancer Registry and Statistics, Cancer Information Service, National Cancer Center, Japan (2019) Cancer mortality data based on demographic statistics 1958–2017. Published May 2019. https://ganjoho.jp/reg_stat/statistics/dl/index.html. Accessed 2 Aug 2019

[13] Sawaki A, Ohashi Y, Omuro Y (2012). Efficacy of trastuzumab in Japanese patients with HER2-positive advanced gastric or gastroesophageal junction cancer: a subgroup analysis of the Trastuzumab for Gastric Cancer (ToGA) study. Gastric Cancer.

[14] Sawaki A, Yamada Y, Yamaguchi K (2017). Regional differences in advanced gastric cancer: exploratory analyses of the AVAGAST placebo arm. Gastric Cancer.

[15] Ohtsu A, Shah MA, Van Cutsem E (2011). Bevacizumab in combination with chemotherapy as first-line therapy in advanced gastric cancer: a randomized, double-blind, placebo-controlled phase III study. J Clin Oncol.

[16] Swain SM, Im YH, Im SA (2014). Safety profile of pertuzumab with trastuzumab and docetaxel in patients from Asia with human epidermal growth factor receptor 2-positive metastatic breast cancer: results from the phase III trial CLEOPATRA. Oncologist.

