# Supplementary material for: Pertuzumab plus trastuzumab and chemotherapy for Japanese patients with HER2-positive metastatic gastric or gastroesophageal junction cancer: a subgroup analysis of the JACOB trial
Source: Int J Clin Oncol. 2019 Oct 16;25(2):301–11. doi: 10.1007/s10147-019-01558-z (PMC6989577; doi:10.1007/s10147-019-01558-z)
Supplement: Supplementary file 1 — Supplementary file1 (DOCX 49 kb) [file 10147_2019_1558_MOESM1_ESM.docx]

# Electronic supplementary material

**Title: Pertuzumab plus trastuzumab and chemotherapy for Japanese patients with HER2-positive metastatic gastric or gastroesophageal junction cancer: a subgroup analysis of the JACOB trial**

**Authors:** Kohei Shitara^1^ • Hiroki Hara^2^ • Takaki Yoshikawa^3,4^ • Kazumasa Fujitani^5^ • Tomohiro Nishina^6^ • Ayumu Hosokawa^7,8^ • Takashi Asakawa^9^ • Satoe Kawakami^10^ • Kei Muro^11^

**Author affiliations:**

^1^ Gastroenterology and Gastrointestinal Oncology, National Cancer Center Hospital East, 6-5-1 Kashiwanoha, Kashiwa, Chiba 277-8577, Japan ([kshitara@east.ncc.go.jp](mailto:kshitara@east.ncc.go.jp))

^2^ Department of Gastroenterology, Saitama Cancer Center, 780 Komuro, Ina, Saitama 362-0806, Japan ([hirhara@cancer-c.pref.saitama.jp](mailto:hirhara@cancer-c.pref.saitama.jp))

^3^ Department of Gastrointestinal Surgery, Kanagawa Cancer Center, 2-3-2 Nakano, Asahi-ku, Yokohama 241-8515, Japan ([tayoshik@ncc.go.jp](mailto:tayoshik@ncc.go.jp))

^4^ Present Address: Department of Gastric Surgery, National Cancer Center Hospital, 5-1-1 Tsukiji, Chuo-ku, Tokyo 104-0045, Japan

^5^ Department of Surgery, Osaka General Medical Center, 3-1-56 Bandaihigashi Sumiyoshi-ku, Osaka 558-8558, Japan ([fujitani@gh.opho.jp](mailto:fujitani@gh.opho.jp))

^6^ Department of Gastrointestinal Medical Oncology, National Hospital Organization Shikoku Cancer Center, Kou-160, Minamiumemoto-machi, Matsuyama, Ehime, Japan ([nishina.tomohiro.nj@mail.hosp.go.jp](mailto:nishina.tomohiro.nj@mail.hosp.go.jp))

^7^ Department of Gastroenterology and Hematology, Faculty of Medicine, University of Toyama, 2630 Sugitani, Toyama, Toyama 930-0194, Japan ([ayhosoka@med.miyazaki-u.ac.jp](mailto:ayhosoka@med.miyazaki-u.ac.jp))

^8^ Present Address: Department of Clinical Oncology, University of Miyazaki Hospital, 5200 Kiyotakecho Kihara, Miyazaki, Miyazaki 889-1692, Japan

^9^ Clinical Information and Intelligence Department, Chugai Pharmaceutical Co., Ltd, 2-1-1, Nihonbashi-Muromachi Chuo-ku, Tokyo, Japan ([asakawatks@chugai-pharm.co.jp](mailto:asakawatks@chugai-pharm.co.jp))

^10^ Clinical Science & Strategy Department, Chugai Pharmaceutical Co., Ltd, 2-1-1, Nihonbashi-Muromachi Chuo-ku, Tokyo, Japan ([kawakamiste@chugai-pharm.co.jp](mailto:kawakamiste@chugai-pharm.co.jp))

^11^ Department of Clinical Oncology, Aichi Cancer Center Hospital, 1-1 Kanokoden, Chikusa-ku, Nagoya, Japan ([kmuro@aichi-cc.jp](mailto:kmuro@aichi-cc.jp))

**Correspondence:**

Kohei Shitara, Gastroenterology and Gastrointestinal Oncology, National Cancer Center Hospital East, 6-5-1 Kashiwanoha, Kashiwa, Chiba 277-8577, Japan. Telephone: 81-47-133-1111; Fax: 81-47-134-6928; E-mail: [kshitara@east.ncc.go.jp](mailto:kshitara@east.ncc.go.jp)

**Table S1** Post-treatment cancer therapy (ITT population)

| Patients, *n* (%) | Pertuzumab  (*n* = 40) | Placebo  (*n* = 40) |
| --- | --- | --- |
| Total number of patients with ≥ 1 treatment | 28 (70.0) | 31 (77.5) |
| Overall number of treatments | 102 | 114 |
| Taxanes | 25 (62.5) | 27 (67.5) |
| Topoisomerase inhibitors | 18 (45.0) | 17 (42.5) |
| Antimetabolites | 1 (2.5) | 5 (12.5) |
| Platinum | 5 (12.5) | 6 (15.0) |
| Cancer immunotherapies |  |  |
| Pembrolizumab | 0 | 2 (5.0) |
| Avelumab | 3 (7.5) | 0 |
| Nivolumab | 0 | 3 (7.5) |
| Blinded nivolumab | 0 | 1 (2.5) |
| Anti-HER2 antibodies |  |  |
| Trastuzumab | 12 (30.0) | 11 (27.5) |
| Trastuzumab emtansine | 0 | 4 (10.0) |
| Anti-angiogenesis agents |  |  |
| Ramucirumab | 4 (10.0) | 6 (15.0) |

*HER2* human epidermal growth factor receptor 2, *ITT* intention-to-treat
